# Disseminated Toxoplasmosis Presenting With Hemophagocytic Lymphohistiocytosis in a Kidney Transplant Recipient

**DOI:** 10.7759/cureus.108331

**Published:** 2026-05-05

**Authors:** Esosa U Ukponmwan, Jessica B Lapasia, Anshul Bhalla, Rana G Sandhu, Sijie Zheng

**Affiliations:** 1 Nephrology, Kaiser Permanente San Francisco Medical Center, San Francisco, USA; 2 Nephrology, Kaiser Permanente, San Francisco, USA; 3 Transplant Nephrology, Kaiser Permanente, San Francisco, USA; 4 Nephrology, Kaiser Permanente Oakland Medical Center, Oakland, USA

**Keywords:** autosomal dominant polycystic kidney disease, cytokine storm, end-stage kidney disease, hemophagocytic lymphohistiocytosis, immunosuppression, kidney transplant, mycophenolate mofetil, prednisone, tacrolimus, toxoplasma gondii

## Abstract

Hemophagocytic lymphohistiocytosis (HLH) is a serious medical disorder characterized by uncontrolled activation of the immune system, leading to a cytokine storm. It can be triggered by infection in patients with genetic predisposition, immunosuppression, or malignancy. Disseminated *Toxoplasma gondii* infection is a rare but recognized trigger in solid organ transplant recipients, particularly in the presence of donor-recipient serologic mismatch. HLH presents with non-specific clinical, systemic, and laboratory abnormalities. We present a case of a 42-year-old man, with a history of kidney transplantation, who presented with a one-week history of fever, diarrhea, and fatigue, diagnosed with HLH due to disseminated toxoplasmosis.

## Introduction

Hemophagocytic lymphohistiocytosis (HLH) is a rare disorder with approximately one to 225 per 300,000 live births and one in 2,000 hospitalizations in adults [1,2]. HLH is a life-threatening and often fatal medical syndrome characterized by dysregulated activation of cytotoxic T lymphocytes, macrophages, and natural killer cells, resulting in a cytokine storm and multiorgan dysfunction [3,4]. Primary (familial) HLH, which usually occurs in children, is associated with abnormal genetic activation of the immune system [4]. Secondary (acquired) HLH is associated with malignancies, autoimmune conditions, pregnancy, post-solid organ transplant, viral infections, including Epstein-Barr virus (EBV) and cytomegalovirus (CMV), and bacterial infections, including *Mycobacterium tuberculosis*, *Toxoplasma gondii *(*T. gondii*), and *Pneumocystis jirovecii* pneumonia (PJP) [1,5]. Diagnosis requires a high index of suspicion, because HLH mimics sepsis and hematologic malignancies [2,3]. Diagnosis is based on the HLH-2004 criteria and H-score [1,5]. Disseminated toxoplasmosis is the occurrence of *T. gondii* cysts or tachyzoites in more than one organ system [5]. Disseminated toxoplasmosis is a rare but severe complication in transplant recipients, particularly in donor-seropositive/recipient-seronegative (D+/R-) mismatch. Here, we report a case of disseminated *T. gondii* infection presenting as HLH in a kidney transplant recipient and review the relevant literature.

## Case presentation

A 42-year-old man with a history of end-stage kidney disease (ESKD) secondary to autosomal dominant polycystic kidney disease (ADPKD), who received a deceased donor kidney transplant (DDKT) in January 2024, was on immunosuppression with tacrolimus, mycophenolate mofetil (MMF), and prednisone.

Transplant history

The kidney transplant donor was a 25-year-old male, donation after brain death secondary to anoxia. Cytomegalovirus (CMV)-donor was positive, and recipient was negative; Epstein-Barr virus (EBV)-donor was positive, and recipient was negative; *T. gondii*-donor was positive, and recipient was negative at the time of kidney transplantation. The renal allograft was a right kidney with conventional anatomy located in the right lower abdomen. Calculated panel reactive antibody (cPRA) 0%; kidney donor profile index (kDPI) 7%; terminal creatinine 0.96 mL/min/1.73 m^2^. Patient had delayed graft function, requiring one session of hemodialysis for hyperkalemia. Following transplant, patient received standard prophylaxis and monitoring protocol for CMV, EBV, and fungal prophylaxis. *Pneumocystis jirovecii* pneumonia (PJP) and *T. gondii *prophylaxis were administered for six months with trimethoprim-sulfamethoxazole (TMP-SMX).

Clinical presentation

He presented eight months after transplant with one week of malaise, fever, decreased appetite, nausea, non-bloody emesis, diarrhea, and frontal and bilateral maxillary headaches. He was alert and oriented, and was chronically ill-appearing. Respiratory, cardiac, and abdominal examinations were unremarkable except for mild tenderness over the right lower quadrant. Vital signs were normal except for mild tachycardia.

Laboratory studies revealed acute kidney injury (AKI) with serum creatinine of 1.97 mg/dL (baseline: 1.2 mg/dL) and tacrolimus level of 13.2 ng/mL. Complete blood count (CBC) and comprehensive metabolic panel (CMP) were initially unremarkable at presentation, but he developed anemia and thrombocytopenia later during hospitalization. Blood culture, respiratory infectious panel, and stool enteric panel, which included Salmonella species, Shigella species, *Campylobacter coli,* and *Campylobacter jejuni*, were negative. EBV was detected at less than 200/mL, far below the 1,000/mL cutoff, indicating the need for treatment. Cytomegalovirus DNA, Aspergillus antigen, and Histoplasma antigen were negative; COVID-19 PCR and *Clostridium difficile* testing were negative.

**Figure 1:**
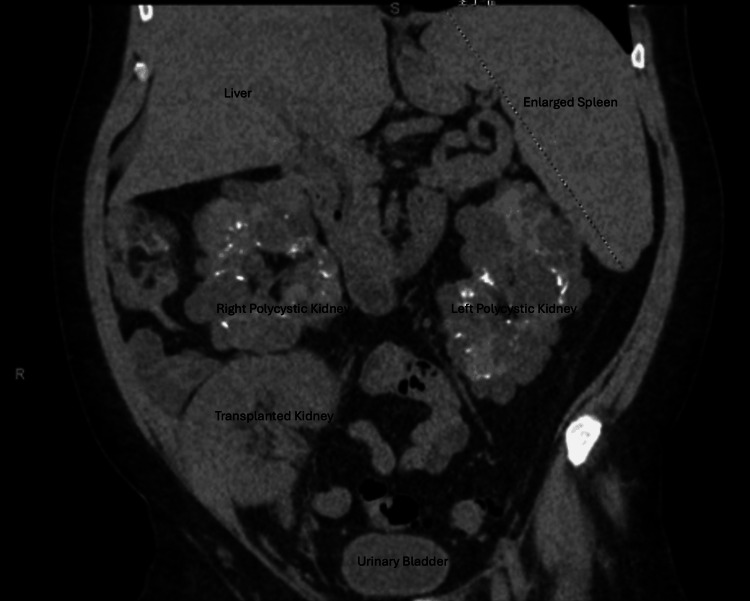
Coronal view of CT scan of the abdomen and pelvis. The image shows the transplanted kidney in the right lower quadrant. Enlarged native kidneys with numerous cysts, moderately enlarged spleen measuring 19 cm in maximal craniocaudal dimension.

**Figure 2:**
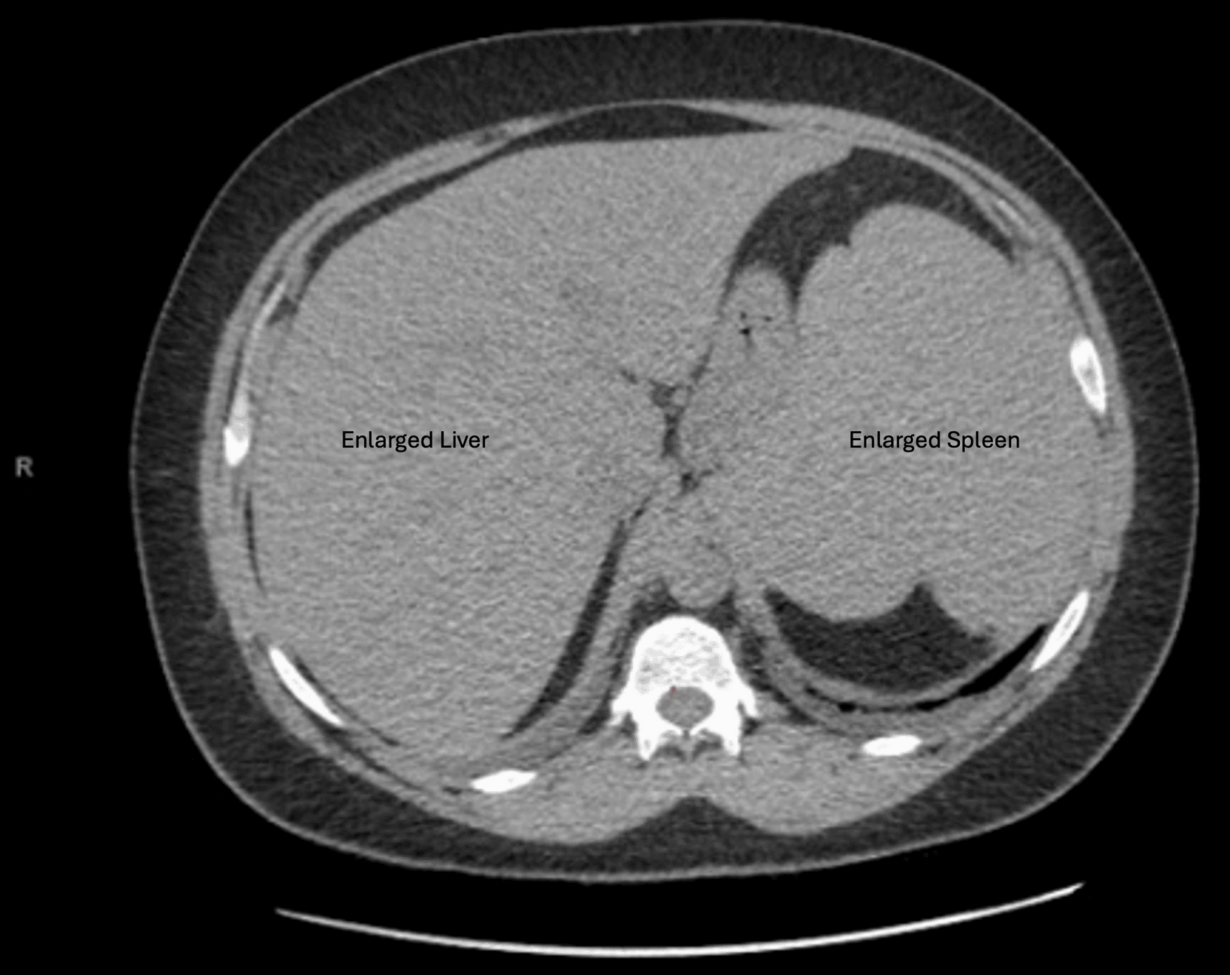
Axial view of the CT scan of the abdomen and pelvis shows hepatomegaly and splenomegaly.

**Figure 3:**
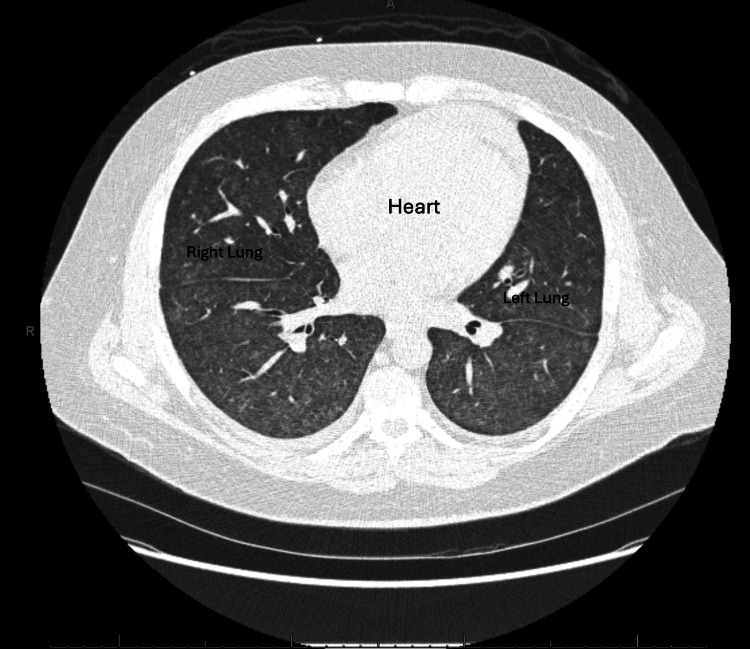
Axial view of the CT scan of the chest shows bilateral ill-defined ground-glass opacities.

**Figure 4:**
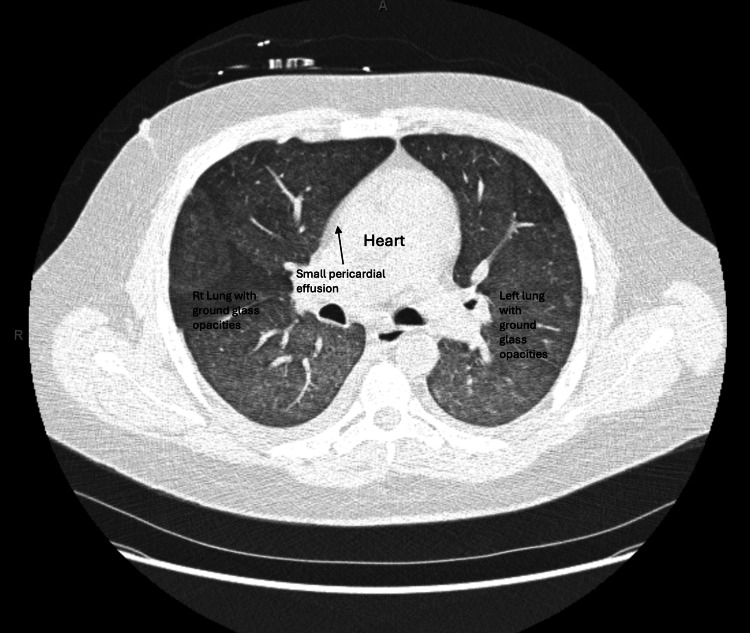
Axial view of CT scan of the chest. The image shows trace pericardial effusion and bilateral ground-glass lung opacities.

Ultrasound scan and Doppler of the transplanted kidney were unremarkable. Computed tomography of the abdomen revealed bilateral polycystic native kidneys and a moderately enlarged spleen (Figures 1, 2). CT scan of the chest revealed bilateral ill-defined ground-glass opacities, small right and left pleural effusions, small pericardial effusion, and coronary artery calcifications (Figures 3, 4). CT scan of the head and neck revealed no abnormalities.

Differentials and hospital course

Our differentials at the time of admission were sepsis and T-cell lymphoma due to hepatosplenomegaly on imaging. The patient was initially treated empirically for sepsis with antibiotics and non-traumatic acute kidney injury. On day two of admission, he became febrile, tachypneic, and tachycardic and received broad-spectrum antibiotics, including ceftriaxone, doxycycline, metronidazole, and vancomycin, given his immunocompromised status. Acute kidney injury was assessed as due to acute tubular necrosis in the setting of sepsis, and no transplant kidney biopsy was done. He received intravenous fluid resuscitation and supportive care. There was concern about post-transplant lymphoproliferative disorder given organomegaly noted on the CT scan.

On day three of admission, a splenic biopsy was performed due to suspicion of post-transplant lymphoproliferative disorder, which showed atypical cytotoxic T-cell infiltrates with concern for hepatosplenic T-cell lymphoma in the setting of post-transplant immunosuppression. Flow cytometry performed on peripheral blood and cerebrospinal fluid was also concerning for T-cell lymphoma.

On day six of admission, the patient remained febrile, developed altered mental status, tachycardia, tachypnea, and acute respiratory failure requiring supplemental oxygen. Antibiotics were changed to alternate-day levofloxacin. His immunosuppression medications, mycophenolate mofetil and tacrolimus, were discontinued due to concern for post-transplant lymphoproliferative disorder, and the prednisone dose was increased. On day nine, his clinical status continued to decline, and he developed multiorgan dysfunction with septic shock and required ventilator support, vasopressor support in the intensive care unit, and continuous renal replacement therapy. The antibiotic regimen was broadened to include piperacillin-tazobactam, atovaquone, and fluconazole.

**Figure 5:**
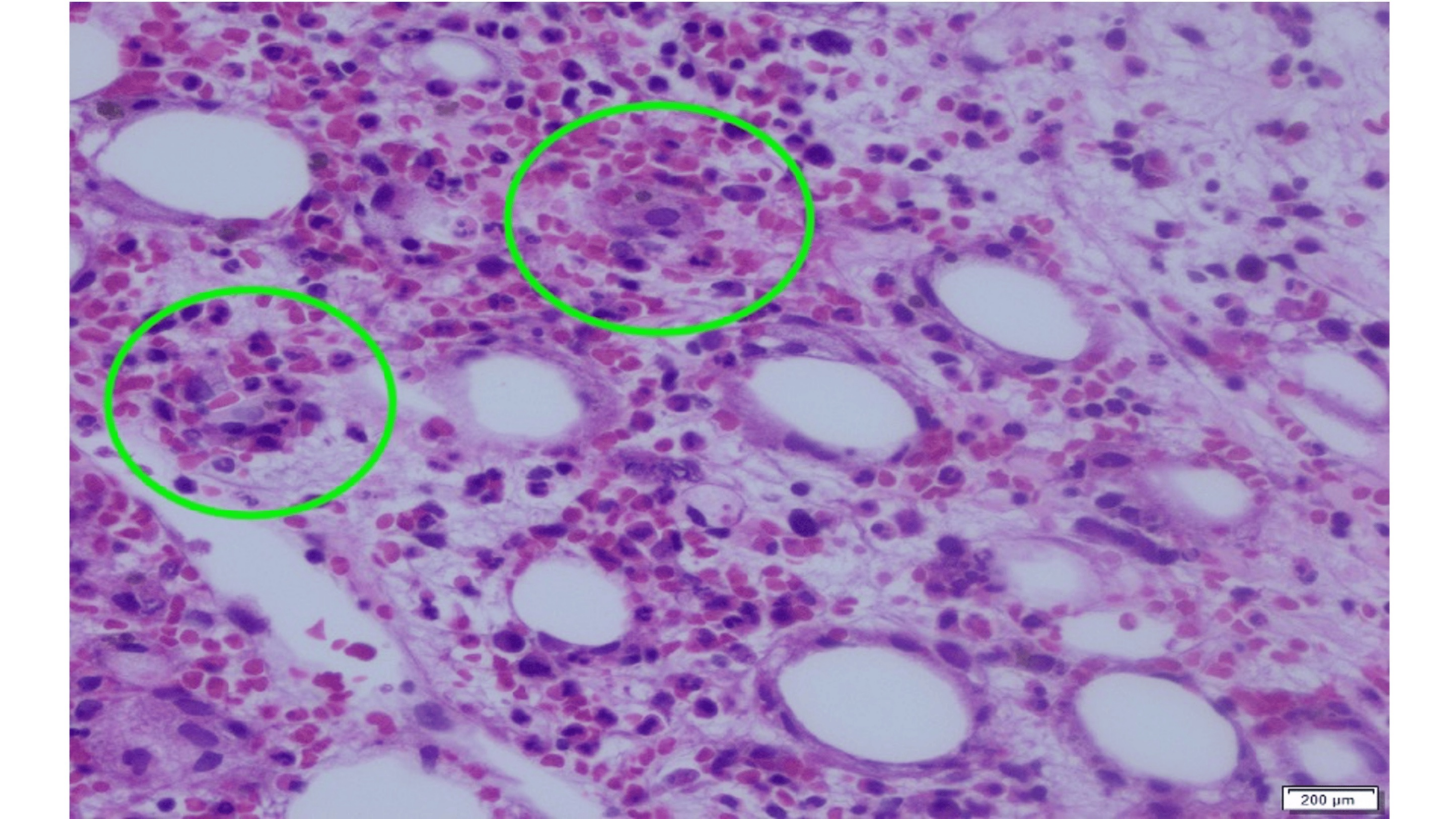
Bone marrow biopsy showing hemophagocytes. Bone marrow biopsy (hematoxylin and eosin stain) showing hypocellular (30%) bone marrow. Markedly increased hemosiderin-laden macrophages with hemophagocytosis (green circle).

On day 10, results showed marked hyperferritinemia (108,329 ng/mL), which raised suspicion for HLH. White blood cell count was 7,300/µL, hemoglobin 9.3 g/dL, and platelets 102,000/µL. Bone marrow biopsy revealed hemophagocytosis (Figure 5). Next-generation quantitative sequencing (Karius test) detected greater than 316,000 molecules per microliter (MPM) of *T. gondii* in plasma. The analytical range of the Karius test is 10-316,000 MPM. This was confirmed by qualitative polymerase chain reaction (PCR) positivity for *T. gondii* in cerebrospinal fluid and bronchoalveolar lavage fluid. He fulfilled six of eight HLH-2004 diagnostic criteria (fever, splenomegaly, bicytopenia, hypertriglyceridemia/hypofibrinogenemia, hemophagocytosis, and hyperferritinemia), with an H-score of 306, indicating >99% probability of HLH. Ophthalmological dilated fundus examination showed no evidence of intraocular *T. gondii*.

The patient was diagnosed with HLH secondary to disseminated *T. gondii* infection. Bone marrow biopsy and peripheral fluorescent in-situ hybridization study were negative for post-transplant lymphoproliferative disorder (PTLD). The HLH-94 protocol was started on day 10 with intravenous etoposide 150 mg/m^2^ for two doses three days apart, and intravenous dexamethasone 10 mg/m^2^ daily for two weeks, followed by an eight-week taper. Prednisone was stopped. Disseminated *T. gondii* infection was treated with sulfadiazine 1,500 mg every 6 h, pyrimethamine 200 mg loading dose, then 75 mg daily, and leucovorin 25 mg daily for an eight-week intensive course, followed by lifelong maintenance therapy. He received acyclovir 400 mg two times daily for viral prophylaxis and posaconazole 300 mg daily for fungal prophylaxis. Relevant laboratory results and trends are shown in Table 1 below.

**Table 1:** The relevant laboratory results during admission and post-discharge. AST: aspartate aminotransferase; ALT: alanine aminotransferase; LDH: lactate dehydrogenase; EBV: Epstein-Barr virus; eGFR: estimated glomerular filtration rate

Day of admission	Laboratory Parameter	Results	Reference range
Day 1	White blood cell	4300/μL	4000 -11000/μL
	Hemoglobin	11.6 g/dL	13-17.0 g/dl
	Platelet	142,000/μL	140-400,000/μL
	Neutrophil	39%	40-60%
	Lymphocytes	10%	20-40%
	Eosinophils	2%	1-4%
	Blood urea nitrogen	23 mg/dL	6 – 20 mg/dl
	Creatinine	1.97 mg/dL	0.6-1.3 mg/dl
	Estimated GFR	42mL/min/1.73m2	>60mL/min/1.73m2
	AST	23 U/L	10-40 U/L
	ALT	24 U/L	0-47 U/L
	LDH	310 U/L	<270 U/L
	Tacrolimus level	13.2ng/ml	5-20 ng/ml (goal: 6-8ng/ml)
	Epstein-Barr virus	<200 IU/ml	Not detected
	Urine protein	100 mg/dL	<29 mg/dL
	Urine hemoglobin	Small	Negative
	Urine appearance	Cloudy.	Yellow
	Urine Specific gravity	1.028	1.001-1.035
Day 10	Ferritin	108,329 ng/ml	22-365ng/ml
	Fibrinogen	211 mg/dl	209-504mg/dl
	White blood cells	7300 /μL	4000 -11000/μL
	Hemoglobin	9.3 g/dl	13-17.0 g/dl
	Platelet	102,000/μL	140-400,000/μL
Day 12	Ferritin	32,313 ng/ml	22-365 ng/ml
	Fibrinogen	120 mg/dl	209-504 mg/dl
Day 13	Ferritin	24,659 ng/ml	22-365 ng/ml
	Triglyceride	892 mg/dl	18-150 mg/dl
	Fibrinogen	164 mg/dl	209-504 mg/dl
Day 14	Ferritin	13,959 ng/ml	22-365 ng/ml
	Triglyceride	614 mg/dl	18-150 mg/dl
Day20	Ferritin	3063 ng/ml	22-365 ng/ml
Day 21	Ferritin	2763 ng/ml	22-365 ng/ml
	EBV	936 IU/ml	Not detected
Day 26	Ferritin	2800 ng/ml	22-365 ng/ml
Day 70(Post discharge)	Ferritin	2314 ng/ml	22-365 ng/ml
	Creatinine	0.91	0.6-1.3 mg/dl
	eGFR	>60mL/min/1.73m2	>60mL/min/1.73m2
	White blood cell	7600/uL	4000 -11000/μL
	Hemoglobin	9.7 g/dL	13-17.0 g/dL
	Platelets	213,000/μL	140-400,000/μL
	Urine Protein/Creatine ratio	0.58 g/g	<0.15 g/g

Outcome

Following therapy, the patient’s clinical status improved, and laboratory values normalized. Encephalopathy, respiratory failure, lymphocytosis, and acute kidney injury (AKI) all resolved. On day 26, ferritin was down to 2,800 ng/mL, and he was discharged home. Four weeks after discharge, he started on reduced-dose tacrolimus 0.5 mg twice daily and resumed prednisone 5 mg daily at the end of an eight-week dexamethasone taper. Tacrolimus was increased to 2 mg twice daily. Eight weeks post-discharge, repeat Karius test showed persistent positivity for toxoplasmosis. Subsequently, he developed biopsy-confirmed Toxoplasma myositis, requiring escalation of anti-parasitic therapy. Maintenance doses of sulfadiazine were increased to 1,500 mg every 6 h, pyrimethamine 75 mg daily, and leucovorin 25 mg daily. Patient’s overall clinical status improved, and he has remained stable.

## Discussion

Primary (familial) HLH in children is usually genetically inherited in a homozygous or heterozygous pattern, causing elimination of the function of cytotoxic and natural killer T-cells [2]. Secondary (acquired) HLH is caused by infections, malignancies, rheumatologic disorders, or drug hypersensitivity reactions [2]. HLH caused by autoimmune or rheumatologic disorders is called macrophage activation syndrome (MAS) [1,2].

Patients usually present with fever, chills, fatigue, headache, nausea, vomiting, diarrhea, and musculoskeletal pain mimicking sepsis syndrome. Diagnosis involves extensive clinical and laboratory investigations, including infectious, hematological, serologic, bone marrow biopsy, and imaging studies [2,6]. The HLH-2004 criteria can confirm HLH when five of eight criteria (fever, splenomegaly, bicytopenia or pancytopenia, hypertriglyceridemia and/or hypofibrinogenemia, hemophagocytosis, low/absent natural killer cell (NK-cell) activity, hyperferritinemia, and high soluble interleukin-2 receptor levels) are met [4,7].

The H-score is calculated based on nine variables (three clinical {known underlying immunosuppression, fever, organomegaly}; five biologic {hypertriglyceridemia, hyperferritinemia, serum glutamic oxaloacetic transaminase and low fibrinogen levels, cytopenia}, and one cytologic {hemophagocytosis on bone marrow aspirate}) to suggest a high probability of HLH with a H-score of 169, each criterion is assigned a value based on logistic regression, with a total score range from 0 to 337 [2,8]. H-score has a sensitivity of 93% and specificity of 85% [2,4,8].

Our patient met six of eight HLH-2004 criteria (fever, bicytopenia, hypertriglyceridemia and/or hypofibrinogenemia, hemophagocytosis, hyperferritinemia, and splenomegaly), and his H-score was 306 points, with a >99% probability of HLH. Elevated ferritin >500 ng/mL is usually observed; ferritin >10,000 ng/mL has 90% sensitivity and 96% specificity for HLH [4,7,8].

Management of HLH is based on the HLH-1994 protocol developed by the Histiocyte Society, using etoposide plus high-dose intravenous dexamethasone tapered slowly over eight weeks, with or without intrathecal methotrexate in patients with neurologic symptoms [1,2,6]. The modified HLH-2004 protocol added cyclosporine A to prevent relapses by opposing the action of Interferon-gamma [2,6]. The 2004 protocol recommended adding intrathecal prednisone in addition to intrathecal methotrexate in patients with progressive neurologic symptoms and performing a human stem cell transplant in patients as soon as a donor is available [1,2]. Management of acquired HLH also involves treating the underlying cause, continued supportive therapy, and close follow-up due to high relapse rates.

Review of literature revealed a few cases of HLH following renal transplant due to *T. gondii*. A case report by Segall et al. describes a kidney transplant recipient who developed invasive toxoplasmosis 10 days after a kidney transplant and hemophagocytic syndrome after receiving a *T. gondii*-positive allograft [9]. Blood cultures and urine cultures were negative. Serum sickness secondary to thymoglobulin was suspected; thymoglobulin was stopped, and high-dose steroids were given. Toxoplasma blood smear, alveolar fluid, and renal allograft biopsy were negative; CT scans of the brain and abdomen were unremarkable; hence, *T. gondii *was not initially diagnosed. The diagnosis of HLH and *T. gondii* was confirmed by a late bone marrow biopsy done on day 20, and the patient died on day 21 of septic shock [9].

Karras et al. identified two cases of HLH secondary to *T. gondii* among 17 patients following renal transplant [10]. Eight of the 17 patients died (47%), and four of the nine surviving patients required graft nephrectomy. Both cases due to *T. gondii* were associated with severe clinical presentations and poor outcomes, including death [10].

Francí et al. reported two cases of donor-transmitted *T. gondii* in kidney transplant recipients who received kidney allografts from the same deceased donor [11]. The first patient was Toxoplasma IgG seronegative at transplant and developed fever on day nine, which was treated with broad-spectrum antibiotics. HLH was suspected on day 25, and serum ELISA, serum, and plasma PCR detected *T. gondii* on day 30. The patient improved with trimethoprim-sulfamethoxazole (TMP-SMX) and a reduction in immunosuppression; he was discharged on day 48. The second patient, who was Toxoplasma IgG seropositive at the time of transplant, developed fever on day 30, with elevated serum ferritin and lactate dehydrogenase; his Toxoplasma serum PCR was positive, and he responded to TMP-SMX and reduction of immunosuppression, and was discharged 10 days after admission [11]. Both cases highlight the risk of transmission of *T. gondii* from donor to recipient during transplant and the difference in severity of illness when there is a donor-recipient (D+/R-) mismatch versus no mismatch [11].

Gay et al. described a case of disseminated toxoplasmosis with HLH in a kidney transplant recipient, confirmed by molecular and serological investigations, following donor/recipient mismatch. The patient developed disseminated *T. gondii* on day 76 with positive PCR in blood, bone marrow, and induced sputum. The patient did not receive *T. gondii*-specific prophylaxis following transplant, but received TMP-SMX for PJP prophylaxis, which was changed to monthly aerosolized pentamidine isethionate on day 10 due to hepatic cytolysis with elevated alanine aminotransferase [12]. The patient improved on treatment with clindamycin and pyrimethamine for six weeks, and subsequent prophylaxis was with atovaquone [12].

Francí et al. presented a case of donor-derived toxoplasmosis with HLH in a kidney transplant recipient, highlighting the risk in high-risk mismatch (D+/R-) settings [13]. These cases demonstrate that toxoplasmosis-induced HLH in kidney transplant recipients is rare but well-documented. They demonstrate a relatively rapid onset of disseminated *T. gondii* and HLH within less than 90 days following transplant, when no prophylaxis was given. They also highlight the poor outcomes, including loss of allograft and death, if HLH is not promptly identified and adequately managed. A summary of the case reports described above is shown in Table 2.

**Table 2:** Summary of case reports on HLH showing the number of patients, time to diagnosis, cause of HLH, and outcome. HLH: hemophagocytic lymphohistiocytosis; CMV: cytomegalovirus; EBV: Epstein-Barr virus; PJP: *Pneumocystis jirovecii *pneumonia; HHV: human herpes virus

Studies	No. of patients	Delay (days)	Cause of HLH	Outcome
Segall et al. [9]	1	10	T. gondii	Died
Karras et al. [10]	17	52	2 due to *T. gondii *and PJP	8/17 died
			9 due to CMV, EBV, HHV6, and HHV8	4/9 survivors required graft nephrectomy
			2 due to tuberculosis and *Bartonella henselae*	
Francí et al. [11]	2	25 and 30	T. gondii	Patients survived; grafts survived
Gay et al. [12]	1	76	T. gondii	Patient survived; graft survived
Francí et al. [13]	1	9	T. gondii	Patient survived; graft survived

The present case highlights that although the risk of transmission of toxoplasmosis in donor-recipient mismatch is low in kidney transplant patients, there is still a risk after completion of six months of prophylaxis with TMP-SMX [14]. Literature review reveals a low risk of less than 1% transmission in kidney transplant, less than 20% risk in liver transplant, and greater than 50% risk of transmission in heart transplant patients [14]. Our patient developed disseminated *T. gondii* eight months following a kidney transplant, after completing six months of prophylaxis with TMP-SMX. Although our patient received the recommended *T. gondii* prophylaxis, disseminated *T. gondii* can present beyond the initial six-month period and requires a high degree of suspicion for *T. gondii* D+/R- patients. A case series and review by Renoult et al. revealed a fatality rate of 64.5% in untreated toxoplasmosis [15].

Our case highlights and demonstrates the use and importance of next-generation sequencing (Karius test) for identifying disseminated *T. gondii *in blood. The present case highlights the advantages of an integrated healthcare system, which include increased access to services and specialists, enhanced information sharing between providers, improved efficiency, and reduced redundancy of tests, which ultimately improved patients’ outcomes. The diagnosis of toxoplasmosis should be suspected in a renal transplant patient with unexplained fever and neurologic symptoms [9]. Prompt recognition and rapid initiation of anti-Toxoplasma therapy with immunosuppression reduction are critical to improving patient outcomes.

## Conclusions

Our case highlights the difficulty associated with the presentation and diagnosis of HLH. It also highlights how HLH mimics other serious medical conditions. Diagnosis requires a high index of suspicion and aggressive management once it is diagnosed to prevent morbidity and mortality. Blood cultures and kidney biopsy may be initially negative, and the diagnosis is often found on serology and bone marrow biopsy. Late diagnosis often leads to poor outcomes and death. Management of HLH involves a multidisciplinary team approach to achieve optimal outcomes. Management also involves treating the underlying cause if identified, supportive management, and close follow-up. Relapse is common within a year of the initial acute illness; patients need close follow-up, and routine monthly laboratory investigations are important for at least the first year after initial diagnosis to achieve optimal outcomes.
